# Effects of ketogenic diet therapy in outpatient anti epileptic drug cost reduction and parental satisfaction among children with intractable epilepsy

**DOI:** 10.3389/fneur.2026.1872749

**Published:** 2026-07-17

**Authors:** Achmad Rafli, Setyo Handryastuti, Amanda Soebadi, Dara Ninggar Santoso, Indira Rahmawati, Ryan Pramana, Rahadian Ramadhan, Clifford Peter Anthony, Irawan Mangunatmadja

**Affiliations:** Department of Child Health, Faculty of Medicine Universitas Indonesia, Dr. Cipto Mangunkusumo Hospital, Jakarta, Indonesia

**Keywords:** children, cost reduction, drug-resistant epilepsy, ketogenic diet, parental satisfaction

## Abstract

**Introduction:**

Epilepsy is a global neurological disorder that contributes to a significant disease burden in children and adolescents. About 11 million children worldwide suffer from epilepsy. Most children with epilepsy respond to anti-epileptic drugs and have controlled symptoms. However, drug-resistant epilepsy (DRE) happens in some children which presents a lot of challenges to their quality of life and development. Alternative treatment for DRE is ketogenic diet therapy (KDT) which is also given alongside the regiment.

**Methods:**

This study aims to compare the prescribed anti-epileptic drug cost of patients receiving KDT compared to the initial pre-diet cost. This study was a descriptive analytical retrospective using outpatient medical records in Dr.Cipto Mangunkusumo General Hospital (RSCM Kiara) from 2021 to 2022. Subjects were patients with drug-resistant epilepsy and underwent a ketogenic diet up to 6 months and aged between 5–18 years old. These patients were then evaluated for their anti epileptic drug costs before starting and after they did a ketogenic diet for up to 6 months with cost evaluation at 3 months interval. The cost of prescribed outpatient antiepileptic drugs were adjusted according to prices set by the Indonesian Ministry of Health. Parents of participants were also surveyed about their satisfaction with KDT using Google Form questionnaire with consent prior to survey.

**Results:**

In this study, a comparison between the overall cost of incorporating KDT for 6 months and pre-diet treatment plan was found to have a statistically significant cost reduction for the incorporation of KDT which demonstrated 21.8% overall reduction in outpatient anti epileptic drug costs among 16 patients which was found to be Rp 3,757,812 or 235 US$ (*p* = < 0.001).

**Discussion:**

This study demonstrated that incorporating KDT for 6 months provides significant financial savings in treatment cost for children with drug-resistant epilepsy.

## 1 Introduction

Epilepsy is a global neurological disorder problem that contributes to a significant disease burden in children and adolescents. About 23 million people in Asia suffer from epilepsy, including children and adolescents (1). Children with epilepsy have uncontrollable seizures due to impulse disturbance in brain activity. The underlying mechanisms of epilepsy are hyperexcitability and repeated synaptic connection between the hyper-synchronized neurons that can be caused by multiple factors such as imbalance of ion concentration, oxidative stress, and brain neurochemical alterations (2). These phenomena creates disturbance between excitation and inhibition equilibrium in the brain. The incidence of epilepsy varies between developed and developing countries. It’s almost threefold higher in developing countries (81.7 per 100.000) compared to developed countries (45 per 100.000) (3, 4). This condition refers to availability of medical services, socioeconomic conditions, and traditional beliefs regarding the treatment of epilepsy. Recent study showed that the maximum incidence of epilepsy was reported 102 per 100.000 cases-year in the age range from 1 to 12 years. It dropped to 24 per 100.000 cases-year in children aged 11–17 years (5).

The majority of children with epilepsy respond to anti-epileptic drugs and have controlled seizures. However, some children do not respond adequately for current treatment and become drug-resistant epilepsy (DRE). According to the 2011 International League Against Epilepsy (ILAE), drug-resistant epilepsy is defined as the persistent of seizures despite at least two anti-epileptic drugs (AED) that are tolerated, properly selected and used (6). Approximately, 25–30% children with epilepsy develop drug-resistant epilepsy which requires alternative treatment for DRE in order to lower the cost of intense anti epileptic drug therapy (7, 8). One such option that is being extensively studied is ketogenic diet therapy (KDT). The Ketogenic Diet (KD) is a high-fat, low-carbohydrate and protein nutritional regimen used to treat DRE. The mechanism of KD to reduce epileptic seizures is still incomplete. Some theories describe that KD reduces glucose levels, increases ketone bodies in blood and cerebrospinal fluid. This state changes the neural metabolism, neural excitability, mitochondrial function and energy reserves. Recent studies and reviews have highlighted important roles of neuro-inflammation and modification of intestinal microbiome which form the basis of ketogenic diet approach with up to 50% seizure reduction following the diet (9–11). In a retrospective cohort study, 58.3% of 24 children treated with KDT showed improvement of 50% in seizures after 6 months of KDT and 33% never experienced another seizure (12).

The major concern about ketogenic diet therapy in the treatment of DRE is the cost. A retrospective study in Memphis, United States (2002), reported comparison of pre-diet, initiating and post diet costs. The total cost used for the treatment of 15 children were, $352,820.20 for the pre-diet period, $41,221.91 for diet initiation, and $149,436.86 for the post-diet period. The study concluded that a successful ketogenic diet provides financial benefits (13). In Asia and especially Indonesia, there is a scarcity of studies about comparing the cost of treatment for children with drug-resistant epilepsy before and after a ketogenic diet therapy initiation. Therefore, this study aims to compare the cost of treatment for children with drug-resistant epilepsy before and after 6 months initiation of ketogenic diet in a developing country and limited resource setting.

## 2 Materials and methods

**Figure 1:**
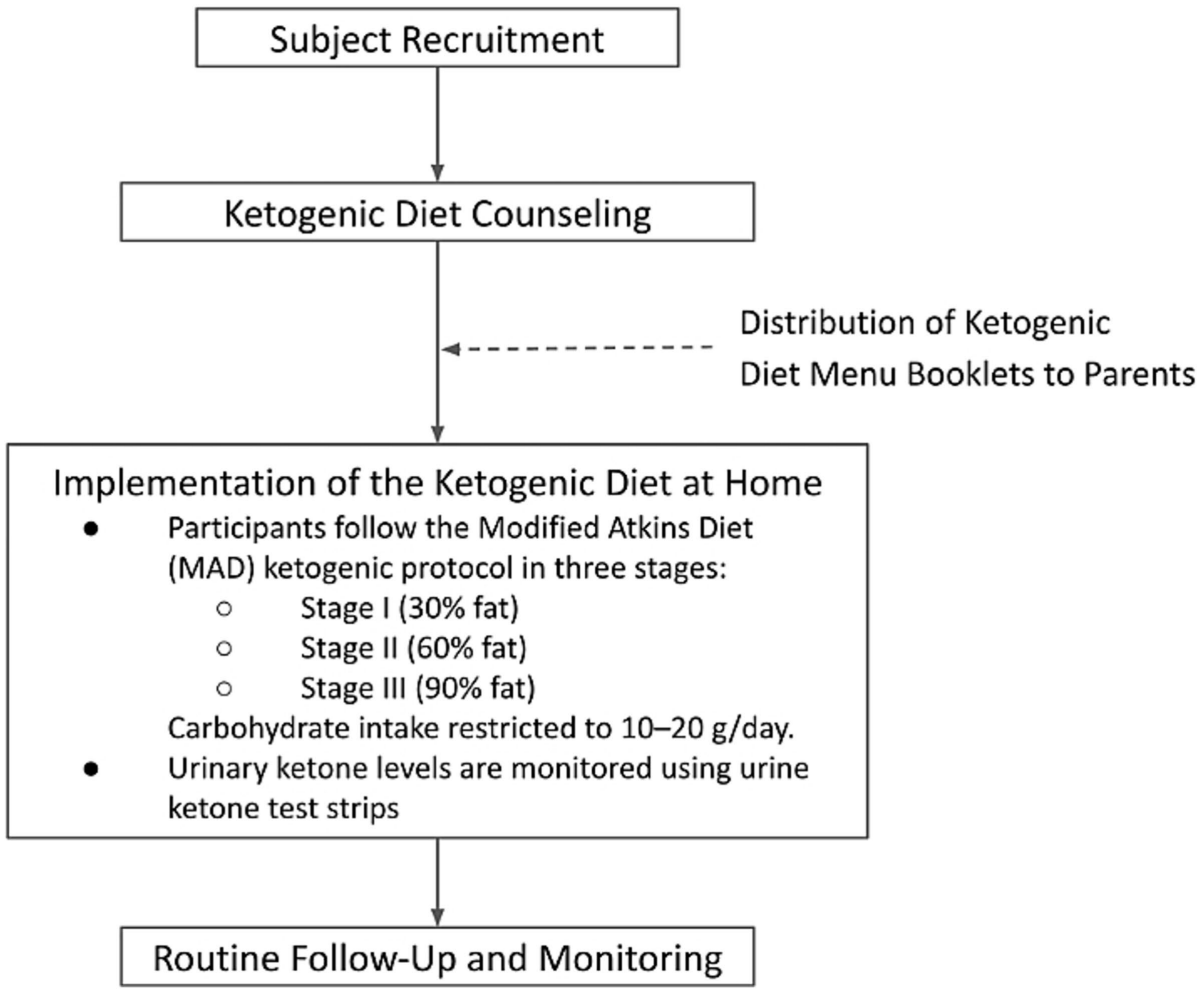
Ketogenic diet flowchart.

This study was a single center descriptive analytical retrospective using outpatient medical records in Cipto Mangunkusumo Hospital Kiara from January 2021 to December 2022. Subjects were patients with intractable epilepsy who also underwent an outpatient ketogenic diet therapy up to 6 months and aged between 2–18 years old whose parents are also willing to be surveyed in regards to parental satisfaction during KDT with exclusion criteria of children with drug resistant epilepsy accompanied by other comorbidities and/or neurological disorders. The type of KDT used in this study was the Modified Atkins Diet (MAD), which was implemented at home by modifying the patients’ daily meal plans with different proportions of fat at each stage, as shown in Figure 1, (9). Parents were also given a booklet of recipes for meals that can be prepared at home and have been adjusted using ingredients that are common and affordable in Indonesia.

These patients were then evaluated for their medical costs before starting a ketogenic diet and after they did ketogenic diet for up to 6 months. Costs for these patients were calculated from the amount of prescribed antiepileptic drugs which were obtained from hospital data. The cost of ketone and vitamin testing is borne by the patient. Sixteen children with intractable epilepsy who underwent a ketogenic diet were included in this study. The cost of antiepileptic drugs was adjusted according to prices set by BPJS and Health Ministry of Indonesia. Total costs for each phase, including initial and 3 until 6 months post-diet, were summed and compared. The parents and legal guardians of the participants are also surveyed and interviewed with a questionnaire consisting of 6 questions describing their experiences and expectations about ketogenic diet therapy for the management of epilepsy.

All data were analyzed using IBM SPSS 26. Descriptive statistics were conducted to summarize the distribution of categorical variables (demographic information, ketogenic diet duration, and amount of anti epileptic drugs administered). The data are presented as frequencies (n) with percentages (%). Statistical analysis of total AED costs in the ketogenic diet study was performed using a paired *t*-test for normal distributed data and the Wilcoxon test for non-normal distributed data based on Saphiro-Wilk test.

## 3 Results

Total data in this study was collected from 16 participants, 56.25% of them were boys. About 56.25% patients were given 3 combinations of Anti-Epileptic Drugs (AED) in pre-diet and 11 patients (68.75%) in 6 months period of ketogenic diet (Table 1). There were 12 different AED used in this study. Topiramate (24.1%) was the most commonly prescribed AED in both pre-initiation and 3 months period of ketogenic diet therapy (Table 2).

**Table 1:** Patient Characteristics.

Subject characteristics	*N* (%)	Values
Gender		
Male	9 (56, 25%)	
Female	7 (43, 75%)	
Age (Years)		11.16 [5–17]
Ketogenic diet phase		
Phase 1	4 (25%)	
Phase 2	6 (37, 5%)	
Phase 3	6 (37, 5%)	
Total of AEDs consumed before ketogenic diet		
2 AED	2 (12, 5%)	
3 AED	9 (56, 25%)	
4 AED	2 (12, 5%)	
>4 AED	3 (18, 75%)	
Total of AEDs consumed after 6 months ketogenic diet		
2 AED	2 (12, 5%)	
3 AED	11 (68, 75%)	
4 AED	1 (6, 25%)	
>4 AED	2 (12, 25%)	

AED, anti-epileptic drugs.

**Table 2:** Type of anti-epileptic drugs.

AED	Before ketogenic diet initiation	After 3 months of diet	After 6 months of diet
	*n* = 54 (%)	*n* = 52 (%)	*n* = 51 (%)
Phenobarbital	1 (1, 9%)	1 (1, 9%)	1 (1, 9%)
Lamotrigine	1 (1, 9%)	1 (1, 9%)	1 (1, 9%)
Levetiracetam	8 (14, 8%)	9 (17, 3%)	9 (17, 6%)
Topiramate	13 (24, 1%)	13 (24, 1%)	13 (26%)
Valproic acid	15 (27, 7%)	13 (25%)	12 (25, 5%)
Clonazepam	2 (3, 7%)	1 (1, 9%)	1 (1, 9%)
Phenytoin	3 (5, 6%)	3 (5, 8%)	3 (5, 9%)
Carbamazepine	5 (9, 3%)	5 (9, 6%)	5 (9, 8%)
Clobazam	5 (9, 3%)	5 (9, 6%)	5 (9, 8%)
Oxcarbazepine	1 (1, 9%)	1 (1, 9%)	1 (1, 9%)

AED, anti-epileptic drugs.

Based on data analysis, Pre-diet costs were greater than 6 months of ketogenic diet in all patients. Total reported monthly anti epileptic drug costs for 16 patients were Rp. 17.271.575 for the pre-diet cost compared to Rp. 13.513.763 for the 6 months period of ketogenic diet therapy. The maximum and minimum cost before ketogenic diet was Rp 2.382.550 (148.92 US$) and Rp 291.300 (18.32 US$) In addition to that, maximum and minimum cost after 6 months ketogenic diet was Rp 1.778.790 (111.85 US$) and Rp 48.116 (3.03 US$) (Table 3).

**Table 3:** Anti epileptic drug cost calculation.

No	Patient	Cost before diet initiation	Cost after 3 months ketogenic diet	Cost after 6 months ketogenic diet
1	AG	Rp1.137.350	Rp915.050	Rp575.090
2	DP	Rp1.853.710	Rp1.778.790	Rp1.778.790
3	BP	Rp1.434.928	Rp1.051.760	Rp1.051.760
4	FH	Rp291.300	Rp48.116	Rp128.550
5	CA	Rp1.250.270	Rp962.070	Rp962.070
6	JD	Rp1.860.150	Rp1.782.150	Rp1.782.150
7	PT	Rp480.630	Rp458.130	Rp458.130
8	AI	Rp328.715	Rp304.149	Rp391.866
9	SH	Rp1.231.740	Rp1.075.740	Rp975.240
10	AR	Rp709.025	Rp450.905	Rp450.905
11	ZZQ	Rp854.775	Rp525.859	Rp525.859
12	AH	Rp615.680	Rp350.724	Rp350.724
13	MRA	Rp937.222	Rp924.939	Rp924.939
14	MAZ	Rp925.290	Rp860.680	Rp860.680
15	AA	Rp978.240	Rp777.780	Rp777.780
16	NF	Rp2.382.550	Rp1.519.230	Rp1.519.240

The difference in pre-diet and 6 months of ketogenic diet cost was Rp. 3.757.812 (235 US$) or 21.8% reduction from the initial pre-diet treatment costs. The data was normally distributed according to the Shapiro–Wilk test (*p* = 0.059). Therefore, a paired t-test was used to compare costs before and after 6 months of treatment, and reported that the pre-diet cost is significantly greater than post-diet total cost (*p* ≤ 0.001) (Table 4) with large effect size (Cohen’s d = 1.01), which indicating a substantial change in treatment costs.

**Table 4:** Total cost for AED.

Comparison of total cost of Anti-Epileptic Drugs (AED)	Pre-diet initiation ketogenic diet	6 Months ketogenic diet	Difference	*p*
Total cost	Rp. 17.271.575	Rp. 13.513.763	Rp. 3.757.812	< 0.001

Comparison of total cost of Anti-Epileptic Drugs (AED)	3 Months ketogenic diet	6 Months ketogenic diet	Difference	*p*
Total cost	Rp.13.786.072	Rp. 13.513.763	Rp. 272.309	0.465

Cost comparison per 3 month basis reported that the total anti-epileptic drug cost difference between 3 month and 6 month incorporation of ketogenic diet therapy was Rp. 272.309 or 17 US$. These data were not normally distributed according to the Shapiro–Wilk test (*p* ≤ 0.001), therefore, analysis was performed using the Wilcoxon signed-rank test. It was reported that the difference in total AED costs between the 3-month and 6-month periods was not statistically significant (*p* = 0.465), with a small effect size (*r* = 0.19) (Table 4).

Furthermore, this study evaluated the acceptance of ketogenic diet therapy by parents. The majority of parents (87.5%) agree that this ketogenic diet is affordable. Most parents involved in this study (81.25%) reported that their children also responded positively to the changes in ketogenic diet menu, with eggs being the most popular choice of menu (81.5%) followed by butter (31.25%), margarine (25.0%), coconut cooking oil (25.0%), mayonnaise (6.25%), olive oil (6.25%), and coconut milk (6.25%) as the options for the additional fat intake on their ketogenic diet menu. Regarding their expectations, some parents said that the diet had met their expectations (43.75%), while some other parents also expressed uncertainty (37.5%) (Table 5).

**Table 5:** Parents’ acceptability toward ketogenic diet.

Components	N (%)
Affordability of cost	
Yes	14 (87, 5%)
No	2 (12, 5%)
Easy/practical to do	
Yes	16 (100%)
No	0 (0, 0%)
Child liked the ketogenic diet menu	
Yes	13 (81, 25%)
No	3 (18, 75%)
Child’s favorite ketogenic diet menu	
Chicken	1 (6, 25%)
Meat	1 (6, 25%)
Fish	1 (6, 25%)
Eggs	13 (81, 25%)
Added fat menu that child likes	
Margarine	4 (25, 0%)
Mayonnaise	1 (6, 25%)
Butter	5 (31, 25%)
Coconut cooking oil	4 (25, 0%)
Olive oil	1 (6, 25%)
Ketogenic diet met parent’s expectations	
Yes	7 (43, 75%)
No	3 (18, 75%)
Uncertain	6 (37, 5%)

## 4 Discussion

### 4.1 Reduction of anti epileptic drug costs

This is the first study in Indonesia to compare the cost of antiepileptic drugs for patients before and after the initiation of ketogenic diet therapy in children with drug-resistant (intractable) epilepsy. Ketogenic diet therapy is expected to lower treatment costs for intractable epilepsy by having a similar efficacy in preventing seizure occurrences, thereby lowering the needed cost for patients and caregivers as they are not required to take as much medications compared to standard anti-epileptic drug regimens. However, ketogenic diet therapy also presented a challenge for parents and caregivers to accept as carbohydrate or starchy foods is an important part of the typical Indonesian household and many of the foods that adhere to ketogenic diet principles are more expensive than common household food. Several prior studies have reported the efficacy of ketogenic diet therapy in the management of intractable epilepsy; however, studies which measure the economic impact of ketogenic diet therapy in terms of cost reduction and parental perception towards such therapies were limited especially in the Asian population. We utilized two studies that were done in the Netherlands to help compare our findings with a previously established study (14, 15). We defined drug treatment costs as the total costs of anti epileptic medications used by patients (list of medications that are used by the participants are shown in Table 2 while the accumulated treatment costs of individual patients are shown in Table 3). The cost per patient was determined by calculating the total amount of drugs administered and the frequency of administration based on data collected from the Cipto Mangunkusumo Kiara hospital outpatient medical records.

Our findings reported a statistically significant difference of treatment cost in patients who underwent up to 6 months of ketogenic diet compared to the cost reported during pre-diet phase in which the cost difference was noted to be Rp. 3.757.812 or 235 US$ in overall cost comparison of 16 patients (*p* < 0.001) of which it reduces 21.8% of the pre-diet treatment cost. Analysis on the anti-epileptic drug cost comparison between the 3 and 6 months period reported that while there is a difference in the cost reduction of anti epileptic drugs required for the patients which was noted to be Rp. 272.309 or 17 US$, it was not a statistically significant cost reduction upon comparison (*p* = 0.484). A cost analysis study done by Van der Louw et al. on 2019 also reported a statistically significant overall treatment cost reduction in 43 patients receiving ketogenic diet therapy for 3 months in outpatient setting compared to 62 patients receiving ketogenic diet in an inpatient setting with a mean difference of treatment cost between the two settings to be € 5,294 (95% confidence interval, *p* < 0.001) (16). Another study done by Whiting et al. in Canada reported that ketogenic diet therapy reduces the frequency and costs of emergency department and inpatient resources utilization among 166 children. Whiting reported that children enrolled in KDT had a mean decrease in Emergency Department utilization cost of CA$630 [95% CI (249–1,012)] per person per year and inpatient cost reduction of CA$1059 [IQR: 7890; p < 0.001] per person per year (17). This shows that ketogenic diet can help reduce the frequency of visits to the hospital which in turn reduces the amount of medications and resources needed that impacts overall treatment costs.

Conversely, a study done by Kinderen et al. in 2015 and a systematic review done by Wijnen et al. in 2017 both in the Netherlands reported that the incorporation of ketogenic diet therapy in the treatment of intractable epilepsy present an increase of treatment cost in patients receiving ketogenic diet initiation for 4 months with a 12 month follow up period (€ 20,986) compared to patients receiving the usual epilepsy treatment (€15,245) (14, 15). This difference compared to our findings can be explained by several factors such as the Kinderen study protocol included a 5-day admission phase for participants assigned to the ketogenic diet group with additional inpatient lab examinations for ketosis and appointments with nutritionists which contributes in increased treatment costs along with loss of parental productivity also being included as one of the parameters for the study’s cost calculations unlike in our study in which we based the cost based on the amount of AEDs administered (14). The Wijnen and Chan systematic review which also incorporated Kinderen’s study found that Ketogenic Diet and Vagus Nerve Stimulation were not found to be cost effective in a 12 month follow up period compared to epilepsy care as usual with an unfavorable cost to quality adjusted life year ratio (€346,899 and €641,068 per QALY) but stated that the probability of ketogenic diet being cost effective rose to 63% in a 5 year period once patients receive ketogenic diet therapy without the need of hospitalization during the initiation of said diet (15, 18). All these findings and comparisons shows that ketogenic diet therapy has a greater cost reduction impact especially for anti epilepsy medications in outpatient settings compared to inpatient settings as intrahospital lab tests and appointments with dietitian can present an increased treatment costs that can also negatively affect the quality of life for patients with intractable epilepsy.

### 4.2 Parental satisfaction regarding ketogenic diet therapy

Our second finding reported that most parents in the study (87,5%) gave positive remarks about the affordability and ease of ketogenic diet therapy in the management of children with epilepsy with all parents of the study participants (100%) agreeing that ketogenic diet therapy can be done at home with ease. However, a large percentage of parents in the study (37,5%) stated that they are unsure about whether ketogenic diet therapy met their expectations in managing intractable epilepsy with 43,75% of parents agreeing that ketogenic diet therapy met their expectations and 18,75% of parents stating that ketogenic approach did not meet their expectations in the management of epilepsy. Such findings can be explained due to the unfamiliarity of Indonesian parents about ketogenic diet therapy which requires omission of carbohydrates and starch from the daily diet which causes worry among parents that their children may not adjust well to such drastic changes in diet. This is in line with studies done by Orr et al. which reported varying responses about ketogenic diet therapy from 17 caregivers who had experienced on average 25 months of ketogenic diet treatment for their children ranging from 2 months to 98 months. The expectations listed in Orr’s study encompasses seizure control, growth and development, along with gastrointestinal side effects on the children; overall Orr’s study reported that the positive impact of KDT outweighs the negative effects and risks associated with the ketogenic diet approach which is in line with our findings that the majority of parents involved in our study accepted the initialization of KDT with minimal adjustment concerns (19).

A study by Semprino et al. reported that the introduction of ketogenic diet therapy with telemedicine monitoring through Google Form questionnaire and WhatsApp communications during the COVID-19 pandemic provided satisfaction with the majority of the parents that were involved with the study (96.3% parents are satisfied with the monitoring of KDT through telemedicine) which helps in long term monitoring of the physical and mental effects of KDT in the management of intractable epilepsy while also help in reinforcing the reliability of google form questionnaire as a tool to gauge parental experiences and satisfaction levels (20). The strength of our study lies in the statistical significance of the cost difference after KDT which reduces about 20% of total medication costs with strong significance value (*p* = 0.001) which also supports the findings from the Van der Louw study and its applicability in long term outpatient setting in a developing country along with this study which also measure parental satisfaction in determining the impact of ketogenic diet therapy in children with drug resistant epilepsy’s quality of life in the setting of a low resource and developing country. However, our study has several limitations such as the use of retrospective analytical study design, small sample size and the parameters used in determining the overall treatment cost parameter. The first limitation in this study was the analytical study design in which it relied on data from secondary sources in which such data presents the possibility of confounding variables present during treatment which we addressed by including a selection criterion of pediatric patients who underwent a ketogenic diet therapy in an outpatient basis with no additional comorbidity and not having hospitalization during the study duration. The second limitation is the small sample size. The sample size was based on participant data which matched with our inclusion criteria during the study duration which was found to contain 16 patients, larger sample size could provide more robust data for statistical analysis. The third limitation was that the outpatient treatment cost parameter was based cumulatively from the costs of medication, while the data about the utilization of ketogenic diet was based from data from the medical record of which it was based from food recall and parental surveys which may also present a recall bias of which we addressed by including detailed questions for parents about the types of ketogenic foods that has been given to their children during the study duration. Future research about this topic should include additional parameters for treatment costs such as costs for household food replacements in accommodating ketogenic dietary requirements and projected cost of parental productivity loss, as well as comparisons between treatment cost from both inpatient and outpatient settings.

## 5 Conclusion

Our preliminary study about the partial cost analysis (drug cost only) through the incorporation of ketogenic diet therapy in antiepileptic treatment regiments for intractable epilepsy in Indonesian patients have shown that the incorporation of ketogenic diet therapy for intractable epilepsy was associated with reduced in treatment costs in this small cohort. Through the results of this study, it is hoped that a ketogenic diet in patients with epilepsy can not only help reduce the frequency of seizures, but also reduce the financial burden on both families and the government. We hope that this pilot study can be used as a basis to help with further research about the economic and social feasibility of ketogenic diet therapy especially in low-resource or developing regions that can help alleviate the burden of medication prices. Future research about this approach should address the limitations present in our study by utilizing larger sample size along with comparing inpatient and outpatient treatment costs utilizing a prospective study design.

## Data Availability

The original contributions presented in the study are included in the article/supplementary material, further inquiries can be directed to the corresponding author.
